# Malaria case management commodity supply and use by community health workers in Mozambique, 2017

**DOI:** 10.1186/s12936-019-2682-5

**Published:** 2019-02-21

**Authors:** Elizabeth Davlantes, Cristolde Salomao, Flavio Wate, Deonilde Sarmento, Humberto Rodrigues, Eric S. Halsey, Lauren Lewis, Baltazar Candrinho, Rose Zulliger

**Affiliations:** 1 Epidemic Intelligence Service, US Centers for Disease Control and Prevention, 1600 Clifton Road, MS A-06, Atlanta, GA 30333 USA; 2 National Institute of Health, Ministry of Health, Maputo, Mozambique; 3 Field Epidemiology Training Programme, African Field Epidemiology Network, Maputo, Mozambique; 4 United States President’s Malaria Initiative, US Agency for International Development, Maputo, Mozambique; 5 National Community Health Worker Programme, Ministry of Health, Maputo, Mozambique; 6 United States President’s Malaria Initiative, US Centers for Disease Control and Prevention, Atlanta, GA USA; 7 Malaria Branch, Division of Parasitic Diseases and Malaria, US Centers for Disease Control and Prevention, Atlanta, GA USA; 8 National Malaria Control Programme, Ministry of Health, Maputo, Mozambique

**Keywords:** Malaria, Mozambique, Community health workers, Supply chain, Integrated community case management

## Abstract

**Background:**

Community health workers (CHWs) provide preventive care and integrated community case management (iCCM) to people with low healthcare access worldwide. CHW programmes have helped reduce mortality in myriad countries, but little data on malaria supply chain management has been shared. This project evaluated the current composition, use, and delivery of malaria iCCM kit commodities in Mozambique—rapid diagnostic tests (RDTs) and artemether–lumefantrine (AL) treatments—to better tailor existing resources to the needs of CHWs in diverse practice settings.

**Methods:**

Health facilities in Maputo (low malaria burden), Inhambane (moderate), and Nampula (high) Provinces were selected using probability proportionate to the number of CHWs at each facility. All CHWs and their supervisors at selected facilities were interviewed using a structured questionnaire to document experiences with kit commodities. Data were analysed to assess CHW commodity stock levels by province and season.

**Results:**

In total, 216 CHWs and 56 supervisors were interviewed at 56 health facilities. CHWs reported receiving an average of 6.7 kits in the last year, although they are intended to receive kits monthly. One-tenth of CHWs reported receiving kits with missing RDTs, and 28% reported lacking some AL treatments. Commodity use was highest in the rainy season. Stockouts were reported by CHWs in all provinces, more commonly in the rainy season. Facility-level stockouts of RDTs or some AL formulation in the past year were reported by 66% of supervisors. Use of CHW kit materials by health facilities was reported by 43% of supervisors; this was most common at facilities experiencing stockouts.

**Conclusions:**

Variations in geographic and seasonal malaria commodity needs should be considered in CHW kit distribution planning in Mozambique. Improvements in provision of complete, monthly CHW kits are needed in parallel with improvements in the broader commodity system strengthening. The findings of this evaluation can help other CHW programmes determine best practices for management of iCCM supply chains.

**Electronic supplementary material:**

The online version of this article (10.1186/s12936-019-2682-5) contains supplementary material, which is available to authorized users.

## Background

The World Health Organization’s Alma Ata Declaration of 1978 emphasized the importance of primary health care for people worldwide [1]. This declaration inspired many countries to create community health worker (CHW) programmes to better provide medical care to their most hard-to-reach communities [2]. CHWs are residents of the areas they serve and are trained to provide basic preventive services. They have been successful in reducing mortality in underserved communities and increasing overall trust in the medical system [3].

As CHW programmes grew more successful, some countries expanded their role into case management as well as prevention. Under a scheme called integrated community case management (iCCM), CHWs diagnose and treat diarrhoea, malaria, and pneumonia, which are the most common causes of child mortality worldwide. CHWs have been very successful in this area and have been shown to be effective at managing these diseases [4, 5]. The original intention of iCCM was to diagnose and treat only children, but some countries have expanded their target population to include adults.

Although iCCM has been implemented in a myriad of countries, little data sharing occurs and there are no internationally recognized best practice guidelines. In the past few years, interest in collaborating across borders has grown, and partners have begun to develop information repositories and organize international meetings [6–8]. However, shared information is still fragmented and focuses heavily on CHW training, CHW impact, and use of mobile technology [9, 10]. For example, while several resources mention the need for robust supply chains, information on how to best manage them is limited [11, 12]. There is also evidence that the absence of adequate, functioning supply systems affects CHWs’ ability to perform their jobs and compromises their credibility [13]. Furthermore, much of the information that is available about CHW programmes comes from the grey literature rather than from peer-reviewed journals.

Mozambique’s CHW programme was founded in 1978 to provide care to the country’s rural and underserved populations. CHWs (called *agentes polivalentes elementares* in Portuguese) have been providing iCCM in Mozambique since 2010 [14]. Healthcare in Mozambique is delivered primarily by the public sector, which is divided into four tiers of facilities. Level 4 hospitals are located in Mozambique’s three largest cities and manage the nation’s most complex cases. Level 3 hospitals in each province’s capital serve as referral centres for patients from around the province. Level 2 facilities include district, general, and rural hospitals and serve as the referral facilities for each district. Finally, Level 1 facilities are health centres and health posts that provide basic primary care in outlying areas [15]. CHWs are assigned to Level 1 and Level 2 facilities in order to extend the reach of the health care system to areas with poor access to medical care. Depending on the needs of the area, the number of CHWs assigned to a facility can range from one to more than ten, and each CHW is responsible for five hundred to two thousand inhabitants [16].

CHWs in Mozambique are expected to divide their time between provision of preventive and curative health services. They should spend 80% of their time conducting health promotion activities, such as health talks, and the rest of their time providing diverse curative services. For example, CHWs diagnose and treat pneumonia in children under 5 years of age and also manage malaria and diarrhoea cases for all ages [17]. Currently, there are more than 3300 CHWs in Mozambique, and the programme aims to have 7000 trained CHWs providing care by 2019 [18]. The contribution of CHWs to malaria case management has steadily increased, and in 2015, CHWs were responsible for diagnosis and treatment of 588,404 of the 6,418,516 individuals registered as receiving malaria treatment through public health services [19].

CHWs in Mozambique are given a medical kit that contains diagnostic and therapeutic supplies needed to perform iCCM. This kit was introduced in 2010 and includes commodities such as oral rehydration solution for treating diarrhoea and amoxicillin for pneumonia. In 2013, a separate, malaria-specific commodity kit was introduced; this kit includes rapid diagnostic tests (RDTs) and treatment courses of artemether–lumefantrine (AL), an anti-malarial [9]. As AL dosing is weight-based, treatment courses are provided in four formulations: small child (for patients weighing 5 kg to < 15 kg), medium child (15 kg to < 25 kg), large child (25 kg to < 35 kg) and adult (35 kg or more). CHW malaria kits are compiled at a single national warehouse in Mozambique’s capital, Maputo. From there, they are distributed to provincial warehouses and then to district warehouses using a push-based system that is similar for malaria and non-malaria CHW kits. Lastly, these kits are transported to CHWs’ assigned health facilities via provincial health authorities [17, 20]. Of note, kits are not packaged and distributed unless all of the required components are in stock.

Kits are intended to be delivered monthly via a push system; that is, CHWs should receive new kits each month, regardless of whether all the materials in their existing kits have been used. The type and quantity of components in the CHW kits were based on rough estimates of CHWs’ commodity usage made by the Mozambique Ministry of Health. These quantities have not been significantly altered since the initiation of iCCM in Mozambique and are not based on current CHW consumption data or on regional and seasonal variations in disease burden [17]. Currently, the malaria CHW kits contain 175 RDTs, 30 small child-formulation AL treatment courses, 15 medium child-formulation AL treatment courses, 10 large child-formulation AL treatment courses, and 30 adult-formulation AL treatment courses. This composition assumes an average of 175 febrile patients per month with a test positivity rate of 49% (85 treatment courses/175 tests).

CHWs have been critical in extending access to malaria services, but they confront important systems constraints. These include challenges related to appropriateness of and access to commodities, limited supervision, and overburdening of activities. There are frequent reports that CHWs are not receiving their commodity kits and that the kits themselves may not appropriately respond to the community needs. For example, in 2015, the number of reported malaria cases ranged from 48,737 in Maputo City to 1,458,212 in Nampula province, but all CHW kits contain the same quantity of RDTs and AL [21]. Similarly, the test positivity rate in these provinces varies considerably, but CHWs receive the same proportion of RDTs relative to AL. The CHW programme also faces continued challenges with transportation of commodities within country and stockouts of malaria commodities [20]. This compromises the programme’s ability to create CHW kits, since they are only made when all commodities are in stock.

Two surveys have evaluated Mozambique’s CHW programme since its expansion into iCCM [17, 22]. However, both of the evaluations were limited in that they were performed before all CHWs had begun to practice iCCM. These surveys identified weaknesses in CHW supervision and supply chain management. The authors recommended several improvements, including increased logistics training, standardized reporting of commodity usage, and mobile phone applications for electronic record keeping and clinical decision support, but these interventions have not been adopted consistently throughout the country.

This project evaluated the current composition, use, and delivery of CHW medical kits to better tailor existing, limited resources to respond to the malaria case management needs of CHWs in diverse practice settings in Mozambique. The findings of this evaluation may also help other CHW programmes determine best practices for management of their supply chains.

## Methods

### Sampling strategy

The provinces of Maputo, Inhambane, and Nampula were purposively chosen for this evaluation as they reflect different epidemiological zones of Mozambique. Maputo Province is the country’s southernmost province. This province recorded a malaria prevalence of 3% among children 5–59 months old who were tested by RDTs in the combined malaria indicator survey in 2015 [23]. Inhambane is a coastal province in the middle of the country that had a malaria prevalence of 23% among children 5–59 months old in 2015 [23]. Nampula, Mozambique’s most populous province, is located in the country’s north and registered a malaria prevalence of 66% among children 5–59 months old in 2015 [23].

**Fig. 1:**
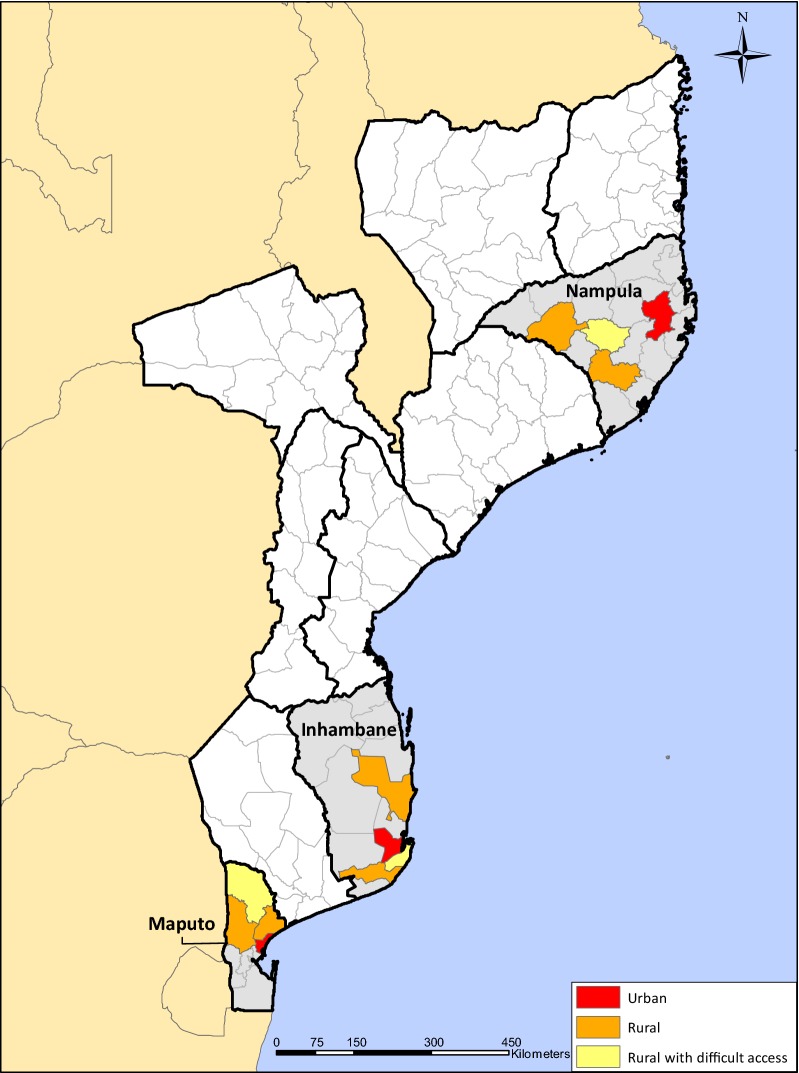
Location of provinces and districts included in Mozambique community health worker malaria commodity kit survey, 2017

Within each of these provinces, four districts were purposively selected to represent a range of urbanization and accessibility: urban areas (one district), rural areas (two districts), and rural areas with particularly difficult access (one district). The districts were categorized by the head of the CHW programme according to standard definitions for urbanization and accessibility used by the Ministry of Health (Fig. 1).

Finally, health facilities in each district were chosen via probability proportionate to the number of CHWs working out of each facility. In order to assess the needs of CHWs in each province with a 10% margin of error and 95% confidence intervals, given a conservative expected outcome proportion of 50%, a minimum sample size of 63 CHWs in Maputo Province, 84 CHWs in Nampula, and 72 CHWs in Inhambane was necessary. A sufficient number of facilities were selected in each province to reach these enrollment numbers, based on the number of CHWs per facility in each district. All CHWs and their supervisors at included facilities were invited to participate in the evaluation.

### Questionnaire

CHWs were interviewed using a standardized questionnaire with open- and closed-ended questions about their use of malaria commodities in the dry and rainy seasons and difficulties encountered with commodity kit delivery. CHWs were asked to estimate the number of kits (both complete kits and any kits missing malaria commodities) they received in the past year. To evaluate commodity use by season, CHWs were also asked to estimate the average number of RDTs and AL treatment courses they used during months they considered to be dry or rainy. In general, Mozambique’s rainy season is from December to April, while the dry season runs from May to November. Additionally, 2 months’ data were abstracted from CHW registers, although these data were not ultimately used for this analysis as many CHWs’ register books were missing or incompletely documented. Supervisors of CHWs were surveyed regarding their opinions of CHW kit stocks and deliveries, health facilities’ use of items from CHW kits, and frequency of health facility-level AL or RDT stockouts. Both groups were also asked to provide their suggestions for improvement of the kit contents and distribution system with open-ended questions. All interviews were conducted in private, and no personally identifying information was collected.

### Data analysis

Data were collected electronically on tablets via the ODK Collect application and were analyzed using SAS version 9.4 (SAS Institute Inc., Cary, North Carolina, USA). For the open-ended questions in the surveys, recurring ideas were identified and grouped into themes that were then coded and used to calculate response frequencies. Open-ended questions addressed respondent suggestions for improving kit components and distribution and where CHWs receive their kits. For closed-ended questions, frequencies and means were calculated. Key analysis indicators were the number of CHWs who were understocked with malaria commodities (examined by province and season), as well as the number of CHWs who received complete kits monthly. Any differences identified between groups were evaluated for statistical significance via the Mantel–Haenszel Chi squared test for frequencies or the two sample T test for means. When comparing across three groups, such as between provinces, results were considered to be statistically significant if the comparison to both other groups was significant.

## Results

### Respondent characteristics

In total, 216 CHWs and 56 supervisors were interviewed in 56 facilities. There were an average of 4.4 CHWs per facility, with the number of CHWs per facility ranging from one to ten. A total of 45 CHWs working in urban areas, 123 in rural areas, and 48 in rural areas with difficult access were included (Table 1).

**Table 1:** Number of community health workers (CHWs) and CHW supervisors interviewed

	Maputo	Inhambane	Nampula	All provinces
Relative malaria burden	Low	Moderate	High	–
CHWs interviewed	68 (31%)	72 (33%)	76 (35%)	216
Urban	24 (35%)	12 (17%)	9 (12%)	45 (21%)
Rural	29 (43%)	40 (55%)	54 (71%)	123 (57%)
Rural with difficult access	15 (22%)	20 (28%)	13 (17%)	48 (22%)
Supervisors interviewed	16 (29%)	26 (46%)	14 (25%)	56

Percentages of the total for each category are in parentheses

### CHW malaria kit delivery and stocks

CHWs reported receiving an average of 6.7 malaria kits per year (Table 2). Only 5% of CHWs reported receiving a kit each month during the past year, and 43% received six or fewer kits. The most common reason for not receiving a kit was that the health facility did not have any CHW kits to distribute (69%). A total of 85% of CHWs reported that they were unable to replenish their supplies if they experienced a stockout before the arrival of their next kit.

**Table 2:** Community health worker (CHW) kit receipt by province, as reported by CHWs in three Mozambique provinces, 2017 (n = 216)

	Maputo	Inhambane	Nampula
Kits received annually, mean	*3.1 (2.6–3.6)*	*8.8 (8.3–9.3)*	*7.9 (7.2–8.5)*
Percent receiving kits monthly	*0.0% (0.0%–0.0%)*	8.3% (1.8%–14.9%)	6.6% (8.8%–12.3%)
Percent receiving six or fewer kits per year	*92.3% (86.3%–99.0%)*	13.9% (5.7%–22.1%)	26.3% (16.2%–36.4%)
Location of kit receipt			
Level 1 health post or health centre	83.8% (74.8%–92.8%)	97.2% (93.3%–100%)	*52.6% (41.1%–64.1%)*
Level 2 hospital	0.0% (0.0%–0.0%)	0.0% (0.0%–0.0%)	*47.3% (35.9%–58.9%)*
CHW’s home	*14.7% (6.1–23.3)*	2.8% (0.0%–6.7%)	0.0% (0.0%–0.0%)
Reasons for not receiving kits			
None at health facility	57.6% (45.3%–69.8%)	68.1% (57.0%–79.1%)	80.3% (71.1%–89.4%)
CHW had commodities remaining	*38.2% (26.4%–50.1%)*	*20.8% (11.2%–30.4%)*	*0.0% (0.0%–0.0%)*
Percent receiving kits with any missing commodities	26.5% (15.7–37.2)	41.7% (30.0%–53.3%)	*57.9% (46.5%–69.2%)*
Missing RDTs	20.6% (10.7%–30.4%)	12.5% (4.7%–20.3%)	*0.0% (0.0%–0.0%)*
Missing AL-small child	19.1% (9.5%–28.7%)	8.3% (1.8%–14.9%)	4.0% (0.0%–8.4%)
Missing AL-medium child	17.7% (8.4%–26.9%)	13.9% (5.7%–22.1%)	23.7% (13.9%–33.5%)
Missing AL-large child	22.1% (11.9%–32.1%)	11.1% (3.7%–18.5%)	9.2% (2.6%–15.9%)
Missing AL-adult	17.6% (8.4%–26.9%)	12.5% (4.7%–20.3%)	14.5% (6.4%–22.6%)
Percent unable to replenish supplies until a new kit arrives	*67.7% (56.2%–79.1%)*	91.7% (85.1%–98.2%)	94.7% (89.6%–99.9%)

Statistically significant values are in italics, and 95% confidence intervals are in parentheses

In general, CHWs in Maputo Province received fewer kits per year, although they were more likely to be able to replenish their kit supplies from other sources. CHWs in Maputo were most likely to get CHW kits delivered to their homes, while CHWs in Nampula were often required to collect their kits from the district hospital rather than from their assigned health facilities. These were all statistically significant differences (Table 2).

Some CHWs also reported that they did not receive new kits because they still had commodities remaining in previous kits, although this goes against the official resupply policy of the Mozambique Ministry of Health. This occurred most frequently in Maputo Province.

A total of 57% of CHWs reported receiving no kits missing malaria commodities within the past year, and 17% reported receiving only one incomplete kit. All formulations of AL were absent from kits at approximately the same rate, with no statistically significant difference between the frequencies of their reported absence from kits (Table 2). However, CHWs in Nampula reported receiving more kits missing RDTs and more kits missing commodities overall, both of which were statistically significant.

Kit delivery data did not differ significantly across levels of urbanization, except that CHWs in rural areas with difficult access were more likely to receive their kits at their assigned health facilities than those in urban or rural areas (Additional file 1: Table S1). Additionally, fewer CHWs in rural areas with difficult access reported not receiving their kits due to lack of kits at the health facilities or due to still having commodities remaining.

### Commodity use

**Fig. 2:**
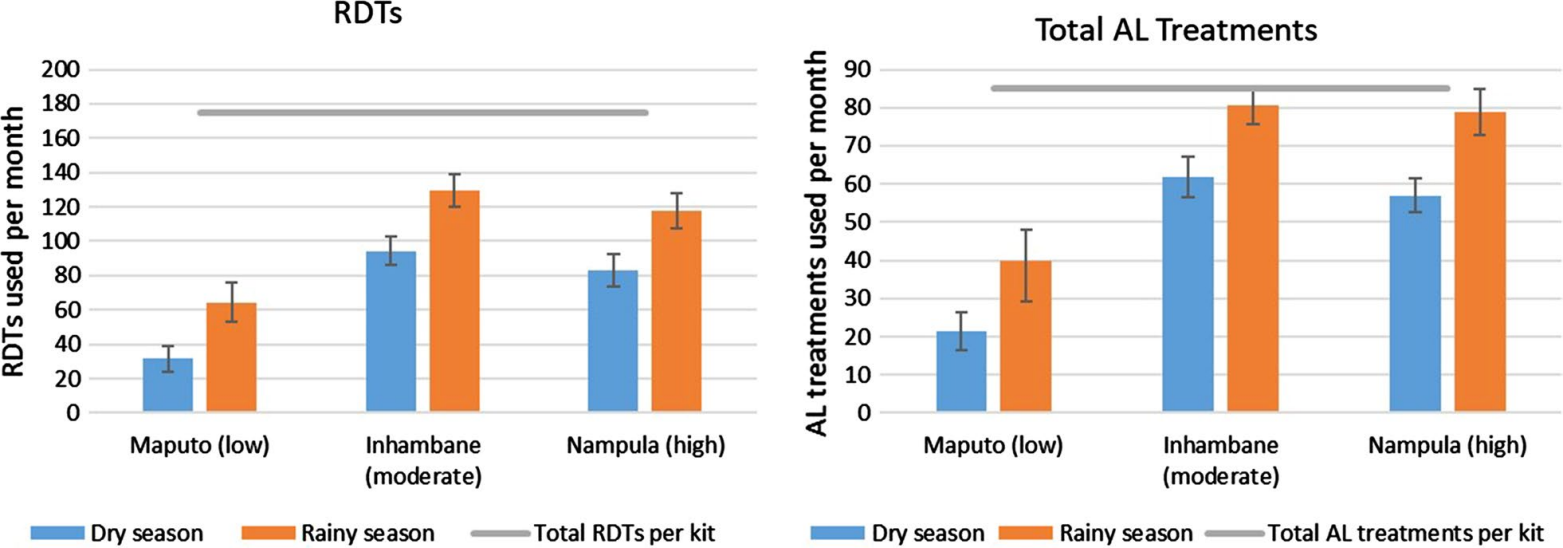
Community health workers’ estimated monthly use of malaria rapid diagnostic tests (RDTs) and artemether–lumefantrine (AL) treatments in the dry and rainy seasons, as compared to the number of RDTs and total AL treatments provided in each kit, by province, 2017 (n = 216). Relative malaria burden of each province is in parentheses, and error bars depict confidence intervals

CHWs in all areas reported increased use of RDTs and AL in the rainy season as compared to the dry season. Commodity use was also increased in provinces with higher malaria burden, with CHWs in Inhambane and Nampula provinces using more than twice as many commodities as CHWs in Maputo (Fig. 2). This difference in commodity use was statistically significant for all commodities in all seasons. CHWs in each province reported use of AL and RDTs at approximately the same rate, regardless of the level of urbanization of their communities (Additional file 2: Figure S1). The only statistically significant difference among these data was that CHWs in rural communities reported more AL use in the rainy season.

**Fig. 3:**
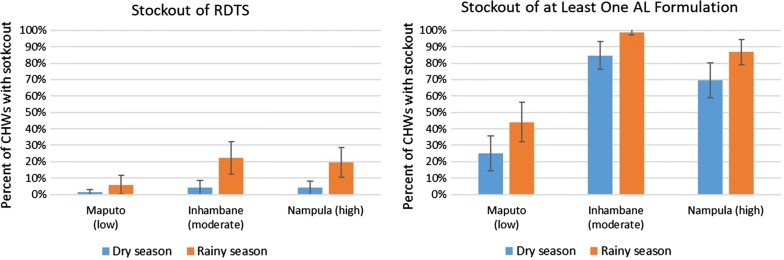
Percentage of community health workers (CHWs) experiencing a stockout of malaria rapid diagnostic tests (RDTs) or at least one artemether–lumefantrine (AL) treatment formulation in the dry and rainy seasons, by province, 2017 (n = 216). Relative malaria burden of each province is in parentheses, and error bars depict confidence intervals

During the months they received kits, 3% of CHWs reported using all the RDTs in their kits during the dry season, and 16% reported this for rainy season. In addition, 61% of CHWs reported stocking out of at least one formulation of AL in the dry season, and 77% reported this in rainy season. Stockouts of both RDTs and AL occurred more than twice as often in Inhambane and Nampula as in Maputo, and this difference was statistically significant for all commodities and seasons (Fig. 3). Results did not differ significantly by level of urbanization (Additional file 3: Figure S2).

**Fig. 4:**
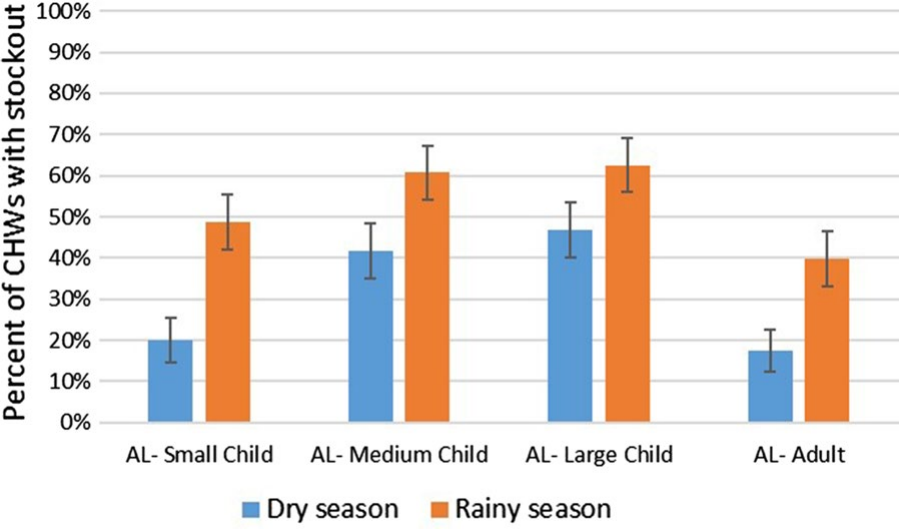
Percentage of community health workers experiencing a stockout of each artemether–lumefantrine (AL) treatment formulation in the dry and rainy seasons in three provinces of Mozambique, 2017 (n = 216). Error bars depict confidence intervals

Treatment courses of AL medium child and large child formulations (which are provided in the current kits in fewer numbers than the AL small child and adult formulations) were most likely to be stocked out (Fig. 4), and this difference was statistically significant for both dry and rainy seasons.

### Health facility stocks

**Fig. 5:**
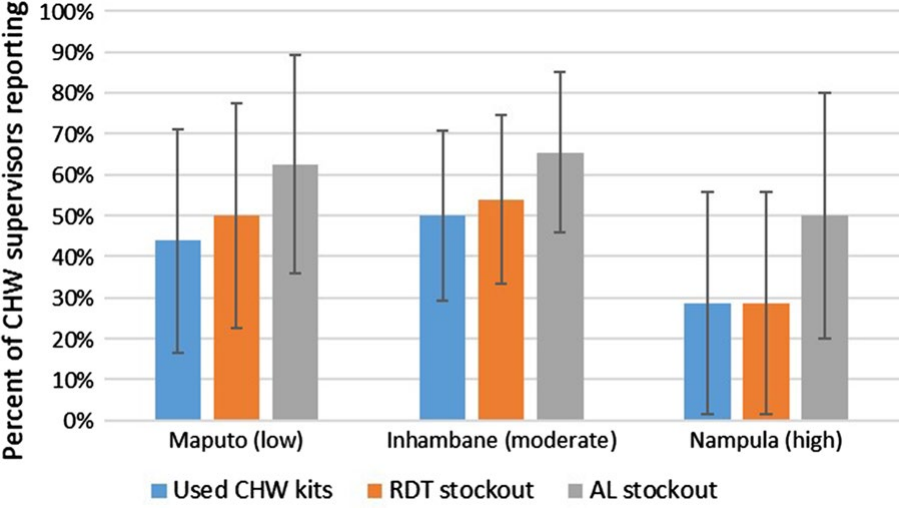
Percentages of community health worker (CHW) supervisors reporting health facility use of materials from CHW kits, health facility malaria rapid diagnostic test (RDT) stockouts in the past year, and health facility stockouts of any artemether–lumefantrine (AL) treatment formulation in the past year, by province, 2017 (n = 56). Relative malaria burden of each province is in parentheses, and error bars depict confidence intervals

A total of 43% of CHW supervisors (n = 56) reported that health facilities had used materials from CHW kits, with 83% of these supervisors stating that this has occurred within the past 6 months. Two-thirds of CHW supervisors reported a health facility stockout of AL or RDTs within the past year (Fig. 5). Stockouts occurred at approximately the same rate across all provinces (Fig. 5) and levels of urbanization (Additional file 4: Figure S3), and no statistically significant differences were detected among these data. Health facilities experiencing stockouts in CHW kit commodities were three times as likely to have used materials from CHW kits. However, confidence intervals were wide due to the small number of CHW supervisors interviewed; the survey was not designed to precisely assess supervisor data.

### Open-ended questions

Sufficient CHWs and supervisors were interviewed to reach saturation for all of the open-ended survey questions. CHWs were asked what they would do if they encountered a patient with possible malaria but had no RDTs. A total of 77% of CHWs stated that they would transfer the patient to the nearest health facility for further management, while 19% would presumptively treat with AL. All CHWs would transfer patients with suspected malaria if they were stocked out of RDTs and AL.

CHWs’ most frequently mentioned complaint regarding the current commodity supply chain was the delivery of kits. A total of 48% of all CHWs and 68% of all supervisors requested that kits be delivered to CHWs’ home communities rather than to their assigned health facilities (Table 3). Of note, CHWs live an average of 18 km from their assigned health facilities, and they receive no specific subsidy for transport. Although kits are supposed to be distributed to CHWs from their assigned health facility, almost half (47%) of Nampula CHWs had to travel to the district hospital to receive their kits.

**Table 3:** Percentage of respondents recommending improvements in community health worker (CHW) kit supply chain, 2017

	CHWs (n = 216)	Supervisors (n = 56)
Deliver kits to CHW communities	48.1% (41.4%–54.9%)	67.9% (55.2%–80.5%)
Deliver kits to health facility	20.4% (15.0%–25.8%)	12.5% (3.6%–21.4%)
Improve resupply consistency	17.6% (12.5%–22.7%)	7.1% (0.2%–14.1%)
Other	2.8% (0.1%–5.0%)	3.6% (0.0%–8.6%)
No changes	16.7% (11.7%–21.7%)	8.9% (1.2%–16.6%)

Respondents could provide more than one recommendation. Confidence intervals are in parentheses

The most common recommendation for improving kit contents was to include more AL treatments (65% of CHWs and 59% of supervisors). Not all respondents recommended specific AL formulations to increase, but of those that did, CHWs placed particular emphasis on medium child and large child AL formulations (28% and 24% of CHWs, respectively). However, 29% of CHWs and 38% of supervisors would make no changes to the current malaria kit composition.

## Discussion

This evaluation of the CHW malaria commodity kit system in Mozambique identified important areas for strengthening, particularly concerning supply chain consistency, stocks within kits, transport of kits, and health facility supplies. Large percentages of CHWs reported commodity stockouts and frequent missed deliveries; without reliable access to malaria supplies, CHWs cannot perform iCCM [13]. For example, CHWs reported referring patients to health facilities when they were stocked out of malaria commodities, thereby introducing transportation costs for community members and, potentially, compromising their own credibility within their communities. Therefore, smoothing the CHW supply chain is an essential part of controlling malaria in Mozambique.

Almost no CHWs in this study received kits as frequently as they were mandated, but kits that did arrive were usually complete. CHWs generally had more RDTs than they could use in 1 month but often ran out of AL treatments. The most frequently mentioned complaint regarding the current commodity supply chain was the delivery of kits, and both CHWs and supervisors requested that kits be delivered to CHWs’ home communities. Finally, almost half of CHW supervisors reported that health facilities had used materials intended for CHWs, and health facilities experiencing stockouts of malaria commodities were more likely to do so.

Mozambique CHWs are intended to receive a new malaria kit every month from their assigned health facility, but this is not occurring in practice. In fact, it appears that the Mozambican supply chain system has adapted a de facto solution to the inadequacies of the CHW malaria kit design for different provincial needs by pushing fewer kits to CHWs in provinces with lower RDT and ACT needs. While this minimizes the problem, it does not adequately address it; this method does not provide the appropriate ratio of RDTs to ACT based on provincial test positivity rates and introduces uncertainty into the commodity system. Studies from sub-Saharan Africa and Southeast Asia have demonstrated that irregular commodity delivery interrupts CHWs’ ability to perform case management. For example, inconsistent medical supplies cause CHW attrition and diminish their credibility within communities [24], while CHWs with less frequent stockouts have higher treatment rates [25].

There is some evidence that inadequate commodity supplies are already affecting CHWs’ iCCM practice in Mozambique. Although the CHW register data collected during this survey was not complete enough for a robust analysis, preliminary analysis demonstrated that there were patients who tested positive for malaria via RDT but did not receive AL treatment. This amounted to 3.5% of RDT-positive children under 5 years of age in the rainy season and 5.9% in the dry season. However, further study is needed to determine if these data are representative of all CHWs and to elucidate the precise causes for not treating RDT-positive cases.

The system for distributing CHW commodities could be adjusted to reflect trends in use and variations in malaria burden. Rather than delivering identical kits at a standard frequency to CHWs nationwide (a push system), CHWs could receive supplies commensurate with their usage (a pull system) [5]. CHW supervisors could use CHWs’ monthly reporting forms to track commodity use and facilitate delivery of additional supplies as needed, a process being used in Rwanda [26]. In Uganda, a set amount of CHW supplies are pushed to the nearest health facility, based on the number of CHWs working out of that facility, and CHWs then pull the materials they need from this pooled supply [27]. CHWs could also monitor their commodity stocks and order more supplies themselves, a strategy that has worked well in Zambia [28]. A similar shift from push to pull has occurred in Mozambique with the health facilities’ malaria commodities, so it may be a feasible solution for CHWs. However, it is important to note that such a system may further compromise CHWs’ access to commodities when health facilities experience commodity shortages.

An additional change that warrants exploration in Mozambique is to formalize a method for CHWs to share supplies with each other or with their assigned health facility, particularly if they possess excess commodities that will soon expire. These approaches have helped streamline CHW supply chains in Kenya, Liberia, and Uganda by making the allocation of supplies more responsive to CHW needs [5, 29]. Furthermore, it is important to coordinate importation of malaria commodities at the national level to increase CHW kit availability downstream [30].

CHW supplies could be tailored to meet their demands even more closely by monitoring their usage in real time and using this data to make programmatic decisions. Usage monitoring recommendations from international CHW workgroups include extending pre-existing logistics management information system resources to the CHW level [31] and using mobile phone applications to track commodity use in real time and anticipate stockouts [6, 7]. In fact, the Mozambican government and its partners have been piloting a mobile health initiative in Inhambane and Cabo Delgado Provinces, using the upSCALE APE CommCare application. This smartphone application provides decision support to CHWs for diagnosis and management of common illnesses and allows for electronic documentation of cases and commodity use [32]. There is an opportunity to use the commodity data generated by this application more extensively as this programme is expanded [33].

It is possible that CHWs may be compensating for current supply shortages by creatively combining their remaining stock—for example, using half of an AL adult dosing formulation to treat a child. This was not directly assessed in the survey, but even if this is occurring, it should not be encouraged. Not only is this method prone to dosing errors, but it is preferable to ensure CHWs have appropriate stocks from the outset, rather than to rely on them making medication adjustments during shortages.

Additionally, CHWs’ reliable and economical access to malaria commodities is essential for their iCCM performance. Reliable provision of transport has been shown to impact CHW effectiveness in Uganda, Senegal, and Bolivia [24, 34]. The most frequent suggestion from both CHWs and supervisors for improving the kit distribution system in Mozambique was to consider delivering CHW kits directly to their communities. This would allow the CHWs to spend more time on treatment and prevention and less time on administrative tasks [24]. Means of achieving this could include sending the kits with traveling community health outreach groups or having the CHW supervisor deliver the kits when monitoring CHWs in their communities. To make transport logistics easier, larger kits could be delivered every other month, rather than smaller kits delivered monthly [35]. An alternative could be to allocate funds for CHWs’ transportation to and from the health facility; in Malawi, for example, CHWs are given bicycles and taught basic bicycle maintenance [36].

Finally, CHW supervisors have indicated that some health facilities keep kit supplies for themselves rather than delivering them to CHWs, and facilities that have experienced a stockout of malaria commodities are more likely to do this. This is an important reminder that any changes to the CHW supply chain must be made in conjunction with improvements to the health facility supply chain.

Assuring more consistent supply of commodities to health facilities has been shown in Ethiopia to reduce the temptation to relieve facility-level stockouts with materials from CHW kits [36]. The WHO recommends periodically evaluating CHW programmes to identify areas for improvement and to monitor progress. The organization also encourages sharing the results with the international community so that other programmes may benefit [24, 26]. To this end, two previous examinations of Mozambique’s CHW programme identified similar issues with the supply chain [17, 22]. Recommendations from these reports, such as collecting better data on CHW commodity consumption, increasing supervision, and employing mobile devices for record keeping, have been implemented in some areas, but room for improvement remains.

This study provides important evidence on areas for continued improvement in the CHW commodity system in Mozambique, but it also highlights areas for further analysis to determine the root cause for such constraints. Key questions for future research include studies to determine the fate of CHWs’ unused supplies, assess the impact of kit stockouts on iCCM practices, and approximate the number of potential malaria patients gone undiagnosed or untreated due to CHW supply chain issues. This is the first evaluation of the CHW malaria supply chain since CHWs began to practice iCCM in Mozambique, and there is much more to be learned about the benefits and drawbacks of the current system. Given the purposeful diversity in selected health facilities by geography, malaria burden, and urbanization, these findings can inform improvements in the national CHW malaria commodity system and may help other CHW programmes who are encountering similar constraints.

### Limitations

This evaluation has some limitations. There was potential for recall bias as much of the analysis relies on CHW and supervisor recollections of commodity use and deliveries. As register data for commodity use and kit deliveries was frequently unavailable, it was not possible to corroborate the estimations provided by survey respondents. The CHW survey did not specify which months of the year were considered the dry or rainy season, leaving the respondents to designate dry and rainy months for themselves; this may make it difficult to translate the results into specific recommended modifications in kit composition by region or month.

Furthermore, CHWs were not asked what they did with any unused supplies, so the ultimate fate of these extra materials is unclear. Some CHWs incidentally reported saving them for use during future stockouts, while others reported that their RDTs and AL had expired, but neither of these issues was directly addressed in the survey. Finally, the non-malaria components in CHWs’ kits were not evaluated during this assessment. These are all important areas for future research.

## Conclusion

This evaluation of Mozambique’s CHW malaria commodity kit system demonstrated that variations in geographic and seasonal malaria commodity needs should be considered in CHW kit distribution planning. Adjustments could be made to provide CHWs with additional antimalarial commodities during the rainy season and in higher malaria burden areas. Additionally, improvements in provision of complete, monthly CHW kits are needed in parallel with improvements in the broader commodity system strengthening.

## Additional files

**Additional file 1: Table S1.** Community health worker (CHW) kit receipt by level of urbanization, as reported by CHWs in three Mozambique provinces, 2017 (n = 216). Statistically significant values are in italics, and 95% confidence intervals are in parentheses.

**Additional file 2: Figure S1.** Community health workers’ estimated monthly use of malaria rapid diagnostic tests (RDTs) and artemether–lumefantrine (AL) treatments in the dry and rainy seasons, as compared to the number of RDTs and total AL treatments provided in each kit, by level of urbanization, 2017 (n = 216). Error bars depict confidence intervals.

**Additional file 3: Figure S2.** Percentage of community health workers (CHWs) experiencing a stockout of malaria rapid diagnostic tests (RDTs) or at least one artemether–lumefantrine (AL) treatment formulation in the dry and rainy seasons, by level of urbanization, 2017 (n = 216). Error bars depict confidence intervals.

**Additional file 4: Figure S3.** Percentages of community health worker (CHW) supervisors reporting health facility use of materials from CHW kits, malaria rapid diagnostic test (RDT) stockouts in the past year, and stockouts of any artemether–lumefantrine (AL) treatment formulation in the past year, by level of urbanization, 2017 (n = 56). Error bars depict confidence intervals.

## References

[1] International conference on primary health care. Declaration of Alma-Ata. WHO Chron. 1978;32:428–30. 11643481

[2] Perry HB, Zulliger R, Rogers MM (2014). Community health workers in low-, middle-, and high-income countries: an overview of their history, recent evolution, and current effectiveness. Annu Rev Public Health.

[3] Tulenko K, Mogedal S, Afzal MM, Frymus D, Oshin A, Pate M (2013). Community health workers for universal health-care coverage: from fragmentation to synergy. Bull World Health Organ.

[4] WHO (2017). Children: reducing mortality.

[5] Perry H, Zulliger R, Scott K, Javadi D, Gergen J, Shelley K, et al. Case studies of large-scale community health worker programs: examples from Afghanistan, Bangladesh, Brazil, Ethiopia, Niger, India, Indonesia, Iran, Nepal, Pakistan, Rwanda, Zambia, and Zimbabwe. USAID/MCHIP; 2017.

[6] iCCM 2014. Lessons learned document. Integrated community case management: evidence review symposium, Accra; 2014.

[7] First international symposium on community health workers: final symposium report, Kampala; 2017.

[8] Iniatives Inc.: CHW Central. 2018. http://chwcentral.org. Accessed 15 Feb 2019.

[9] Kallander K, Strachan D, Soremekun S, Hill Z, Lingam R, Tibenderana J (2015). Evaluating the effect of innovative motivation and supervision approaches on community health worker performance and retention in Uganda and Mozambique: study protocol for a randomised controlled trial. Trials.

[10] Thondoo M, Strachan DL, Nakirunda M, Ndima S, Muiambo A, Kallander K (2015). Potential roles of Mhealth for community health workers: formative research with end users in Uganda and Mozambique. JMIR mHealth uHealth.

[11] Young M, Wolfheim C, Marsh DR, Hammamy D (2012). World Health Organization/United Nations children’s fund joint statement on integrated community case management: an equity-focused strategy to improve access to essential treatment services for children. Am J Trop Med Hyg.

[12] Perry H, Zulliger R (2012). How effective are community health workers?.

[13] Perry H, Zulliger R (2012). How effective are community health workers? An overview of current evidence with recommendations for strengthening community health worker programs to accelerate progress in achieving the health-related Millennium Development Goals.

[14] Chilundo BG, Cliff JL, Mariano AR, Rodriguez DC, George A (2015). Relaunch of the official community health worker programme in Mozambique: is there a sustainable basis for iCCM policy?. Health Policy Plan.

[15] United States President’s Malaria Initiative: Mozambique malaria operational plan FY 2018. United States President’s Malaria Initiative; 2017.

[16] CCM Central: Mozambique. Maternal and child survival program; 2018.

[17] USAID DELIVER PROJECT Task Order 4: Mozambique. Strengthening the community health worker supply chain. United States Agency for International Development; 2012.

[18] MISAU. Programa Nacional de Controlo da Malária: Plano Estratégico da Malária 2017–2022. (Pública DNdS ed. Maputo2017.

[19] Mozambique National Statistics Institute. Weekly epidemiological bulletins, Maputo; 2015.

[20] President’s Malaria Initiative. Malaria operational plan FY 2018, Washington, DC; 2017.

[21] Mozambique Ministry of Health. National malaria control program annual report, Maputo; 2015.

[22] Save the Children: Survey of Agentes Polivalentes Elementares (APEs) providing integrated Community Case Management in Nampula, Mozambique. Save the Children; 2013.

[23] MISAU, INE. Inquérito de Indicadores de Imunização, Malária e HIV/SIDA em Moçambique (IMASIDA) 2015: Relatório de Indicadores Básicos, Maputo; 2016.

[24] Alliance Global Health Workforce (2010). Global experience of community health workers for delivery of health related milennium development goals: A systematic review, country case studies, and recommendations for integration into national health systems.

[25] Oliphant NP, Muniz M, Guenther T, Diaz T, Lainez YB, Counihan H (2014). Multi-country analysis of routine data from integrated community case management (iCCM) programs in sub-Saharan Africa. J Glob Health.

[26] Chandani Y, Andersson S, Heaton A, Noel M, Shieshia M, Mwirotsi A (2014). Making products available among community health workers: evidence for improving community health supply chains from Ethiopia, Malawi, and Rwanda. J Glob Health.

[27] Uganda Ministry of Health. Supply chain system for community health programs in Uganda: a situation analysis. USAID/Uganda Health Supply Chain Program; 2016.

[28] Biemba G, Chiluba B, Yeboah-Antwi K, Silavwe V, Lunze K, Mwale RK (2017). A mobile-based community health management information system for community health workers and their supervisors in 2 districts of Zambia. Glob Health Sci Pract.

[29] Malaria Commodities Program. Strengthening platforms for case management in communities. President’s Malaria Initiative.

[30] John Snow Inc (2017). Quantification of health commodities: a guide to forecasting and supply planning for procurement.

[31] iCCM Task Force Supply Chain Management Subgroup. Tips on supply chain management issues for CCM, Washington, DC; 2015.

[32] Malaria Consortium. Mobile health (mHealth) approaches to improve motivation and performance of CHWs in Mozambique, London; 2014.

[33] Malaria Consortium. upSCALE: mHealth system strengthening for case management and disease surveillance, London; 2016.

[34] Sunguya BF, Mlunde LB, Ayer R, Jimba M (2017). Towards eliminating malaria in high endemic countries: the roles of community health workers and related cadres and their challenges in integrated community case management for malaria: a systematic review. Malar J.

[35] Oliver K, Young M, Oliphant N, Diaz T, Kim J. Review of systematic challenges to the scale-up of integrated community case management: emerging lessons and recommendations from the Catalytic Initiative (CI/IHSS). United Nations Children’s Fund; 2012.

[36] Chandani Y, Noel M, Pomeroy A, Andersson S, Pahl MK, Williams T (2012). Factors affecting availability of essential medicines among community health workers in Ethiopia, Malawi, and Rwanda: solving the last mile puzzle. Am J Trop Med Hyg.

