# Supplementary material for: Malaria case management commodity supply and use by community health workers in Mozambique, 2017
Source: Malar J. 2019 Feb 21;18:47. doi: 10.1186/s12936-019-2682-5 (PMC6385463; doi:10.1186/s12936-019-2682-5)
Supplement: Supplementary file 1 — Additional file 1: Table S1. Community health worker (CHW) kit receipt by level of urbanization, as reported by CHWs in three Mozambique provinces, 2017 (n = 216). Statistically significant values are in italics, and 95% confidence intervals are in parentheses. [file 12936_2019_2682_MOESM1_ESM.docx]

Additional Table 1. Community health worker (CHW) kit receipt by level of urbanization, as reported by CHWs in three Mozambique provinces, 2017 (n=216). Statistically significant values are in italics, and 95% confidence intervals are in parentheses.

|  | Urban | Rural | Rural with Difficult Access |
| --- | --- | --- | --- |
| Kits received annually, mean | 5.8  (4.7-6.8) | 7.2  (6.6-7.8) | 6.2  (5.3-7.1) |
| Percent receiving kits monthly | 6.7%  (0.0%-14.2%) | 5.7%  (1.5%-9.8%) | 2.1%  (0.0%-6.3%) |
| Percent receiving six or fewer kits per year | 57.8%  (42.8%-72.8%) | 36.6%  (28.0%-45.2%) | 45.8%  (31.2%-60.5%) |
| Location of kit receipt |  |  |  |
| Level 1 health post or health centre | 80.0%  (67.8%-92.2%) | 69.1%  (60.8%-77.4%) | *95.8%*  *(90.0%-100%)* |
| Level 2 hospital | 17.8%  (6.2%-29.4%) | 22.8%  (15.2%-30.3%) | *0.0%*  *(0.0%-0.0%)* |
| CHW’s home | 0.0%  (0.0%-0.0%) | 8.1%  (3.2%-13.0%) | 4.2%  (0.0%-10.0%) |
| Reasons for not receiving kits |  |  |  |
| None at health facility | 77.8%  (65.1%-90.4%) | 76.0%  (68.3%-83.7%) | *43.8%*  *(29.2%-58.3%)* |
| CHW had commodities remaining | 13.3%  (30.1%-23.7%) | 10.6%  (5.1%-16.1%) | *45.8%*  *(41.9%-63.1%)* |
| Percent receiving kits with any missing commodities | 31.1%  (17.0%-45.2%) | 46.3%  (37.4%-55.3%) | 43.8%  (29.2%-58.3%) |
| Missing RDTs | 11.1%  (1.6%-20.7%) | 11.4%  (5.7%-17.1%) | 8.3%  (0.2%-16.4%) |
| Missing AL-small child | 8.9%  (0.2%-17.5%) | 11.4%  (5.7%-17.1%) | 8.3%  (0.2%-16.4%) |
| Missing AL-medium child | 11.1%  (1.6%-20.7%) | 23.6%  (16.0%-31.2%) | 12.5%  (2.8%-22.2%) |
| Missing AL-large child | 15.6%  (4.5%-26.6%) | 14.6%  (8.3%-21.0%) | 10.4%  (1.5%-19.4%) |
| Missing AL-adult | 13.3%  (3.0%-23.7%) | 19.5%  (12.4%-26.7%) | 4.2%  (0.0%-10.0%) |
| Percent unable to replenish supplies until a new kit arrives | 68.9%  (54.8%-83.0%) | 91.1%  (85.9%-96.2%) | 85.4%  (75.1%-95.8%) |
