# Supplementary figures and images for: Malaria case management commodity supply and use by community health workers in Mozambique, 2017
Source: Malar J. 2019 Feb 21;18:47. doi: 10.1186/s12936-019-2682-5 (PMC6385463; doi:10.1186/s12936-019-2682-5)

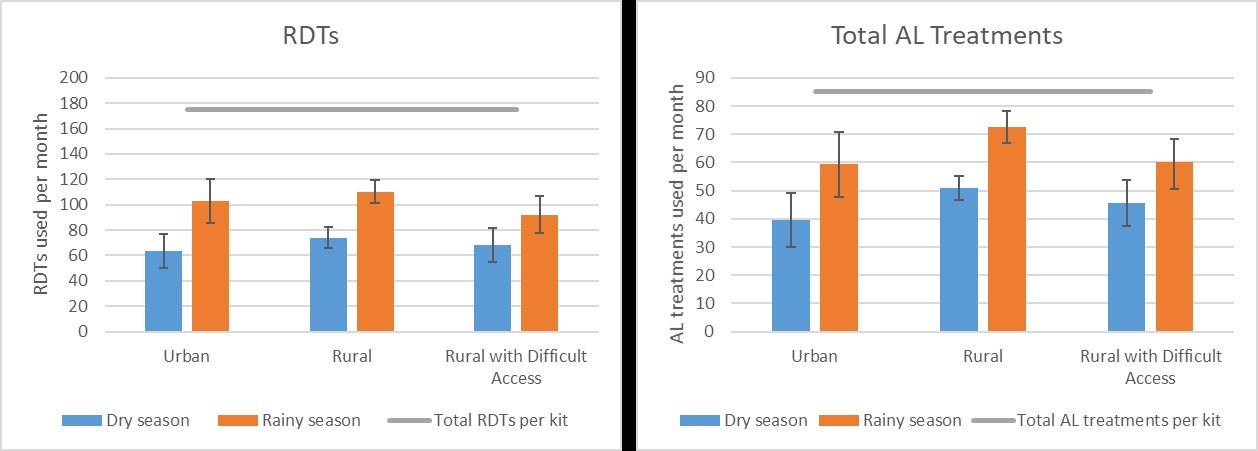

Supplement: Supplementary file 2 — Additional file 2: Figure S1. Community health workers’ estimated monthly use of malaria rapid diagnostic tests (RDTs) and artemether–lumefantrine (AL) treatments in the dry and rainy seasons, as compared to the number of RDTs and total AL treatments provided in each kit, by level of urbanization, 2017 (n = 216). Error bars depict confidence intervals. [file 12936_2019_2682_MOESM2_ESM.jpg]

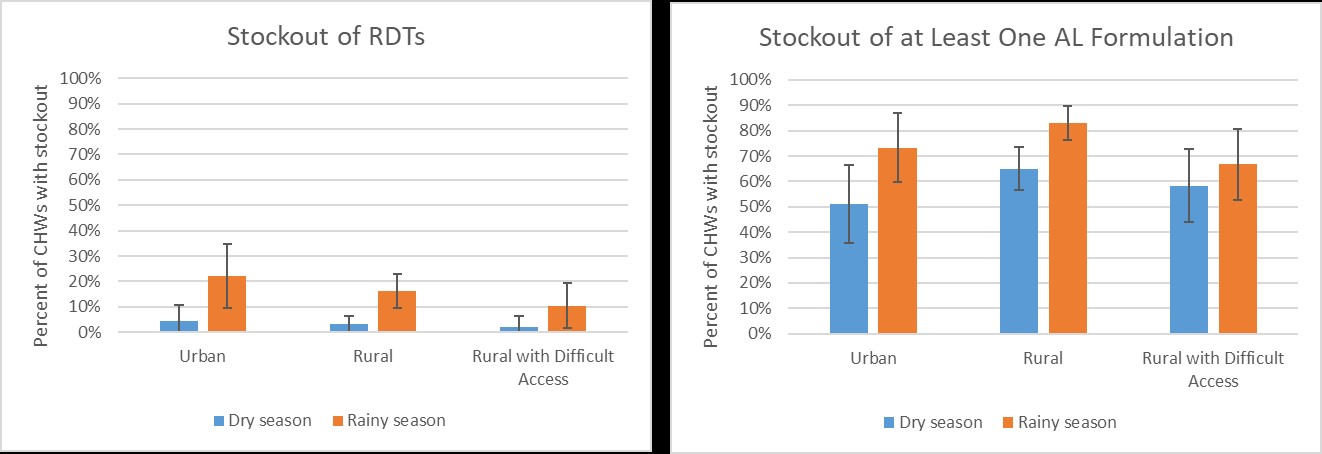

Supplement: Supplementary file 3 — Additional file 3: Figure S2. Percentage of community health workers (CHWs) experiencing a stockout of malaria rapid diagnostic tests (RDTs) or at least one artemether–lumefantrine (AL) treatment formulation in the dry and rainy seasons, by level of urbanization, 2017 (n = 216). Error bars depict confidence intervals. [file 12936_2019_2682_MOESM3_ESM.jpg]

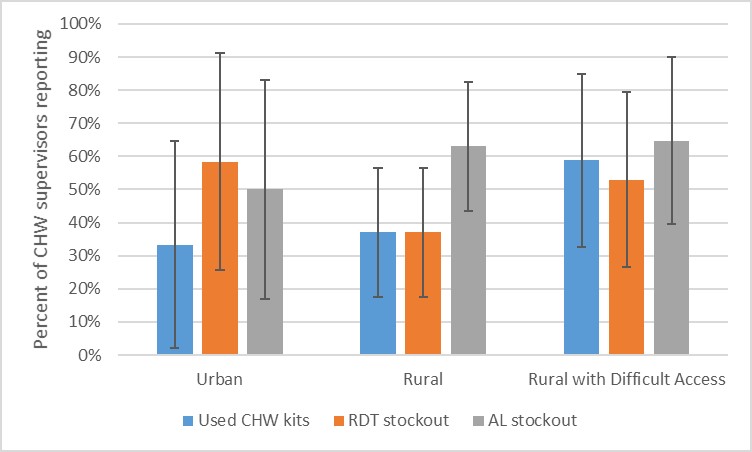

Supplement: Supplementary file 4 — Additional file 4: Figure S3. Percentages of community health worker (CHW) supervisors reporting health facility use of materials from CHW kits, malaria rapid diagnostic test (RDT) stockouts in the past year, and stockouts of any artemether–lumefantrine (AL) treatment formulation in the past year, by level of urbanization, 2017 (n = 56). Error bars depict confidence intervals. [file 12936_2019_2682_MOESM4_ESM.jpg]
